# Collapsing Focal Segmental Glomerulosclerosis in a Patient with Systemic Lupus Erythematosus

**DOI:** 10.1155/2014/732192

**Published:** 2014-08-11

**Authors:** Hassan Tariq, Arsalan Rafiq, Giovanni Franchin

**Affiliations:** Bronx Lebanon Hospital Center, Department of Medicine, 1650 Selwyn Avenue, Suit 10C, Bronx, NY 10457, USA

## Abstract

We present a case of a 36-year-old female from Ghana who presented with atypical chest pain and shortness of breath and was found to have bilateral transudative pleural effusion and trivial pericardial effusion. Further work-up revealed serological markers consistent with active lupus and negative HIV. She developed rapid deterioration of her renal function requiring dialysis. Her renal biopsy showed collapsing focal segmental glomerulosclerosis with diffuse mesangial proliferative glomerulonephritis, consistent with lupus nephritis class II along with tubular degenerative changes. She was started on high dose steroids and later on mycophenolate mofetil. Her renal function slowly recovered to baseline.

## 1. Introduction

Collapsing glomerulopathy is segmental or global wrinkling and collapse of capillary walls and overlying epithelial cell proliferation [1]. When we trace back literature, first cases trace back to 1979 which have been biopsy proven [2]. An increasing number of cases are being reported as awareness among health care professionals is increasing [3]. It is a podocytopathy with distinct clinicopathologic features from focal segmental glomerulosclerosis as the lesions are characterized by formation of pseudocrescents and by collapse of capillary loops rather than extracellular matrix accumulation and glomerulosclerosis being a late manifestation. Three variants have been described in the literature including idiopathic, genetic, and reactive [4]. Collapsing glomerulosclerosis presents with proteinuria, resistant to most available treatments, and leads to rapid progression towards renal failure. The usual causes for this manifestation include viral infections, autoimmune disorders, or drugs. Here we present a case of a patient with collapsing glomerulosclerosis which will provide more insight and further research opportunities in regard to the treatment of this distinct pathologic condition.

## 2. Case Presentation

A 36-year-old black woman from Ghana presented to the ER with complaints of chest pain and shortness of breath for one day. She was returning from a vacation in Ghana when she experienced sudden onset of left-sided pleuritic chest pain during the flight, followed by shortness of breath after landing. She was diagnosed with Sjogren's syndrome when she presented five years earlier with a vasculitic rash of lower extremities which showed leukocytoclastic vasculitis on biopsy. At the time she had positive ANA at 1 : 1280 titer, positive rheumatoid factor, ESR and CRP elevated, anti-double stranded DNA negative, anti-SSA positive, anti-SSB positive, and normal complements. Except for a few recurrent episodes of rash in lower extremities which responded to steroids, she remained asymptomatic for a couple of years prior to the current admission.

She had been recently admitted to a hospital in Ghana after being found to have a left-sided pleural effusion and treated with chest tube insertion for presumed pneumonia. Review of systems and social history were noncontributory.

Upon admission to our institution, she was afebrile and showed stable vital signs. Except for decrease breath sounds at the lung bases and a purulent discharge from site of recent chest tube removal, remaining of physical exam was unremarkable. Blood tests revealed anemia with hemoglobin of 9.2 g/dL, hematocrit of 29.2, white cell count of 3.9 with 46% neutrophils, and a platelet count of 287. Blood urea nitrogen was 11 mg/dL and creatinine 0.6 mg/dL. Chest CT showed a small pericardial effusion and bilateral loculated pleural effusion.

**Figure 1:**
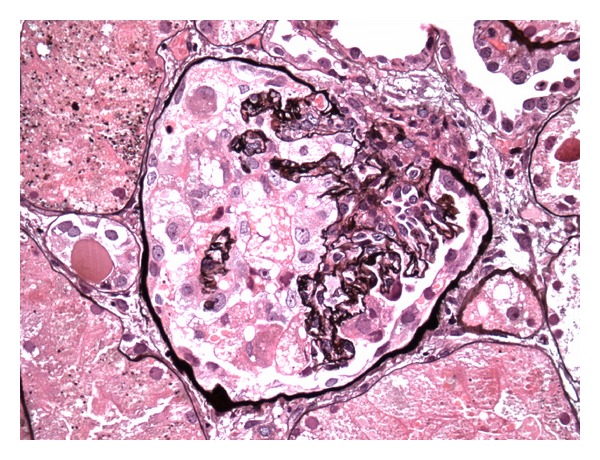
Light microscopy showing collapsing focal segmental glomerulosclerosis with mesangial hypercellularity and resulting collapse of the glomerular capillaries.

**Figure 2:**
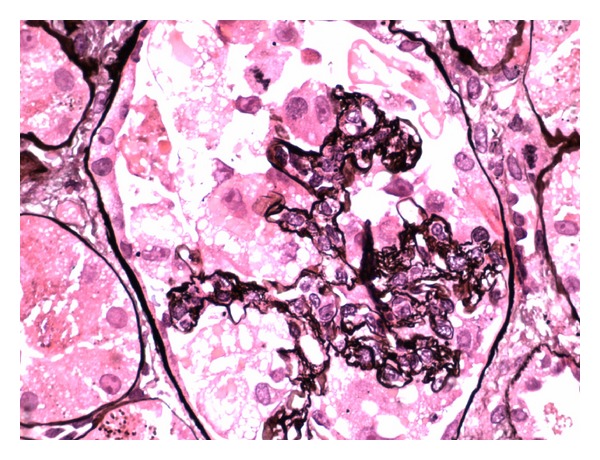
Light microscopy again showing collapsing focal segmental glomerulosclerosis with mesangial hypercellularity and glomerular capillary collapse.

**Figure 3:**
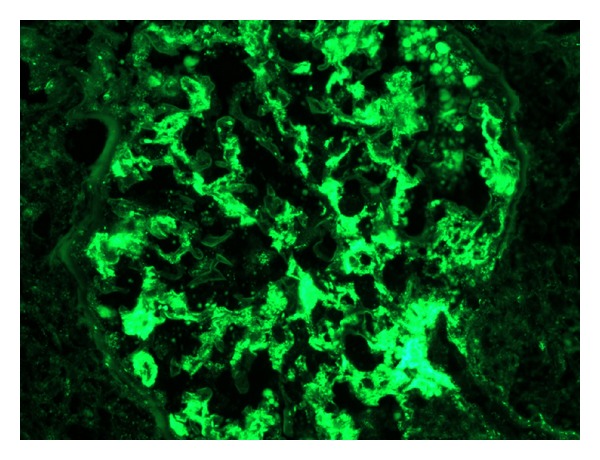
Immunofluorescence reveals granular global mesangial deposits.

**Figure 4:**
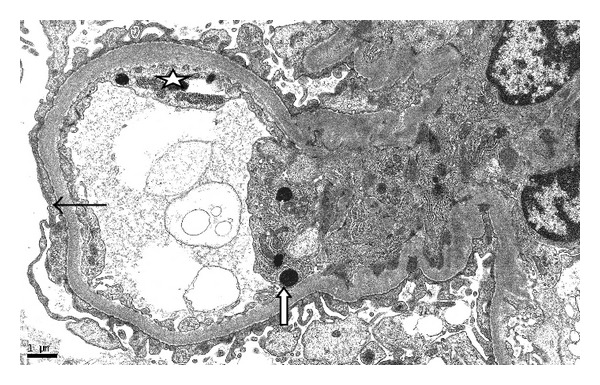
Ultrastructural findings of focal segmental glomerulosclerosis showing podocyte foot process effacement (thin black arrows) and protein reabsorption droplet (white arrow). Endothelial cells show tubuloreticular inclusions in the upper right-hand corner (star).

**Figure 5:**
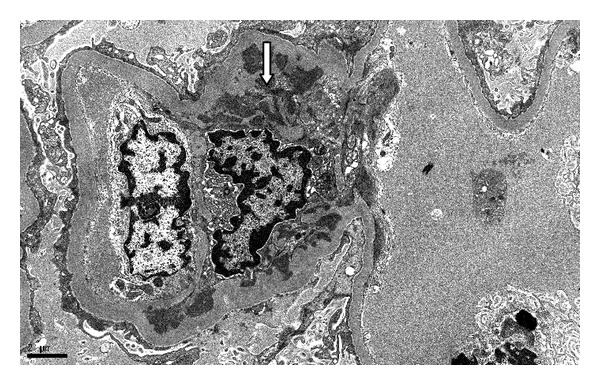
Collapsing lesion of focal segmental glomerulosclerosis with GBM wrinkling and mesangial deposits (arrow).

She was initially treated with a broad spectrum antibiotic regimen of vancomycin and piperacillin-tazobactam for presumed healthcare-associated pneumonia. ANA remained positive at a titre of 1 : 640 while complement levels were low (C3 was 36; C4 was 5). Echocardiogram showed a normal ejection fraction with small pericardial effusion. Prednisone 40 mg and hydroxychloroquine for presumed lupus-induced serositis were started. Her renal function worsened rapidly after day 5 of hospitalization with an increase in creatinine from 1.1 mg/dL to 5.2 mg/dL over 48 hours with further increase up to a peak creatinine of 9.1. Urine showed proteinuria of 5.2 grams/day. Hyaline and granular casts were present in her urine in addition to many RBC and WBC suggesting a component of acute tubular necrosis possibly mixed with lupus-associated nephritis. Patient received pulse doses of 1 g methylprednisolone daily for three days. In view of worsening azotemia and metabolic acidosis, patient was started on hemodialysis. CT-guided pleural fluid drainage was found to be a transudate with negative culture results. Renal biopsy showed 100% effacement of foot processes. Electron dense global mesangial deposits were present with few subepithelial, segmental deposits. Endothelial tubuloreticular inclusions were also seen. Pathology was consistent with collapsing focal segmental glomerulosclerosis (Figures 1 and 2) with diffuse mesangial proliferative glomerulonephritis, consistent with lupus nephritis class II along with tubular degenerative changes. Immunofluorescence revealed granular global mesangial deposits which stain 3+ for IgG, 1+ for IgM, 2+ for IgA, 3+ for C3 and C1, and 3+ for kappa and lambda (Figure 3). On ultrastructural evaluation, 1 of the 4 glomeruli sampled displayed a collapsing lesion of focal segmental glomerulosclerosis with GBM wrinkling and retraction with swelling of overlying visceral epithelial cells which contain protein resorption droplets (Figures 4 and 5). There were no foci of fibrinoid necrosis seen.

Her course was complicated by thrombocytopenia presumably secondary to hydroxychloroquine. She remained on high dose steroids with 120 mg prednisone daily. Her renal function slowly improved to a creatinine of 0.7 with an e-GFR of 122 mL/min/1.73 m^2^ in about 4 weeks. Patient remained with proteinuria of 3.3 grams/24 h. Mycophenolate mofetil was started as prednisone was being tapered and complement levels normalized while both antibodies to double stranded DNA and ANA became negative.

## 3. Discussion

Collapsing glomerulopathy (CG) has a rapid clinical course with massive proteinuria and relative resistance to standard treatments available. It has become an increasingly recognized pattern of glomerular injury with pathologic appearance characterized by global or segmental collapse of the glomerular capillary tuft, with wrinkling and retraction of the capillary walls overlaid by epithelial cell proliferation in the Bowman space that is frequently accompanied by tubulointerstitial disease [2, 5, 6]. CG shares features with HIV induced nephropathy (HIVAN) but has been shown to be a totally separate entity on its own independent of HIV infection [2, 7, 8]. Epidemiologically CG in HIV and non-HIV patients was similar in terms of age, sex ratio serum creatinine, proteinuria, interstitial damage, and extent of glomerular pathological findings [8].

Associations with numerous etiologies apart from HIV infection have been reported including other infections (tuberculosis, CMV, parvovirus B19, and hepatitis B and C), drugs (including pamidronate, anabolic steroids, heroin, and interferons), malignancies (multiple myeloma, hemophagocytic syndrome, and acute monoblastic leukemia), and autoimmune diseases like SLE, adult Still's disease, and mixed connective tissue disease, while in the primary form it is seen mostly in patients of African descent [1, 2, 6, 7, 9–14]. It is more common in females compared to males [5]. It is mostly seen in adults with median age of 30–40 years; however extremes of age occurrences have also been reported [15].

Underlying immune-mediated pathophysiology has been postulated as a causative mechanism. Some studies have also postulated an ischemic pathophysiology involving antiphospholipid antibodies leading to a secondary thrombotic microangiopathy [5, 16]. At the genetic level, idiopathic CG and HIV-associated nephropathy are associated with disappearance of specific podocyte markers (CALLA, WT-1, podocalyxin, GLEPP-1, and C3b receptor) from all collapsed glomeruli and of synaptopodin from 16% of noncollapsed glomeruli and define a dysregulated podocyte phenotype [17]. CG pattern of podocytopathy has been shown to be linked to allelic variants in APOL1 in black patients [13, 18]. Microscopically it is often difficult to differentiate and diagnose CG on renal biopsy especially if there is also evidence of lupus nephritis. However there are certain morphological features that can help like presence of protein reabsorption droplets within the glomerular cells point towards CG while presence of endothelial proliferation with fibrinoid necrosis spindle-cells, fibrin is seen mostly in lupus nephritis. Also seen in CG is that portion of the glomerulus not involved by crescents do not show endocapillary hypercellularity [19]

Patients with CG usually present with severe renal failure, massive proteinuria and usually have a poor response to steroids and other immunosuppressive therapy [2, 6, 13, 20–22]. They may also present with hypoalbuminemia, hypercholesterolemia, and edema with manifestations being not different compared to noncollapsing focal segmental glomerulosclerosis (FSGS) [15]. Compared to CG, patients with noncollapsing focal segmental glomerulosclerosis show some response to steroids and other immunosuppressive therapies [19]. CG has an aggressive course with the mean time from biopsy diagnosis to end-stage renal disease of 13 months compared with 63 months in patients with idiopathic focal segmental glomerulosclerosis [23] suggesting that CG has a more worse prognosis [15, 20, 22]. These patients carry a poor prognosis and most of them end up being dialysis dependent [6], reported to be around 50–100% even with the currently available treatment [3, 24, 25].

There is currently no evidence based therapy for CG and therapeutic options available are derived from empiric approach of treating it as other renal disorders or associated diseases. Recommendations for current treatment are based on regimens used to treat FSGS in non-HIV patients. The overall treatment results are not encouraging with a complete remission rate of less than 10% and a partial remission rate begin reported to be 15.2% [3]. However studies have shown no difference in the overall response to steroid treatment among the histologic variants of FSGS [26]. Treatment options include steroids, cyclosporine, aggressive blood pressure control with angiotensin converting enzyme inhibitors and/or angiotensin II receptor blockers, and lipid lowering agents. Retrospective studies have shown good results when steroids are started shortly after diagnosis, although there are no prospective treatment trials [25]. Role of other immunosuppressants including mycophenolate mofetil is yet to be defined [6, 25]. Some studies have shown plasmapheresis as an option for treatment of recurrent FSGS and it has been shown to decrease proteinuria and glomerular injury [27]. Novel treatment strategies using cyclin-dependent kinase inhibitor CY202 have shown some promise but require more research [28].

Prognostic features have been researched in many studies. In one retrospective study, data from 42 patients with CG and 18 patients with HIVAN showed that end-stage renal disease (ESRD) was increased significantly by interstitial fibrosis of >20%, proteinuria of >8 g/d, creatinine of >2.0 mg/dL, glomeruli with collapsing lesions >20%, and HIV infection [8]. One study found progression to ESRD predicted by serum creatinine concentration at the time of renal biopsy and lack of remission of proteinuria [2]. Another study also found response to therapy as the best prognostic indicator of outcome regardless of the histologic subclassification in adults with primary FSGS [26].
